# Assessing Nursery-Finishing Pig Manures on Growth of Black Soldier Fly Larvae

**DOI:** 10.3390/ani13030452

**Published:** 2023-01-28

**Authors:** Jianwei Hao, Shuang Liu, Aiguo Luo, Jia Zhao, Shengli Shi, Yun Zhang, Chujun Li

**Affiliations:** 1 Department of Biological Science and Technology, Jinzhong University, Jinzhong 030619, China; 2 Institute of Loess Plateau, Shanxi University, Taiyuan 030006, China; 3 The Brain Cognition and Brain Disease Institute (BCBDI), Shenzhen Institute of Advanced Technology, Chinese Academy of Sciences, Shenzhen 518055, China; 4 Department of Entomology, Texas A&M University, 2475 TAMU, College Station, TX 77843-2475, USA

**Keywords:** black soldier fly larvae, fattening pig, manure, hemicellulose

## Abstract

**Simple Summary:**

With the increasing consumption of pork, pig manure has become an important pollution problem in the pig livestock industry. It is worth noting that the amount of manure from fattening pigs far exceeds that from breeding herds. During the fattening period, the manure’s composition may vary depending on the stage of the pig’s growth, from nursery to finishing, and on the variety of feed. The treatment of manure by black soldier fly larvae (BSFL), *Hermetia illucens* (L.), (Diptera: Stratiomydiae) is an emerging waste management technology. Although studies have shown that BSFL could be used in swine manure treatment, there is little literature discussing the nutrient discrepancies of swine manure types and their influences on BSFL growth. Thus, this study analyzed the nutrient composition of different fattening pig manure types. Furthermore, the factors that influence larval growth were identified. This could be used in further waste treatment and formulation diets for BSFL.

**Abstract:**

Livestock manure is an important component of agricultural organic waste, and in recent years, with the development of research on the bioconversion of manure, BSFs have been proven to be useful in the treatment of a variety of livestock wastes. In-depth research on the composition of manure and its effect on the development of BSFL is, however, very scarce. The purpose of this study was to identify the parameters that influenced the growth of BSFL that was fed fattening pig manure. The pH, moisture, and nutrients of the fattening manures (namely, nursery, growing, and finishing pig manures) were measured. To examine the influence of manure types on larval growth, 100 larvae were inoculated in 100 g of each type of manure in triplicate. According to the findings, larvae fed finishing pig manure had the lowest dry weight (30.2 ± 6.1 mg) compared to those fed growing (58.2 ± 7.3 mg) or nursery (65.5 ± 6.2 mg) pig manure. The correlation coefficients (*r*) between the nutrients in the manure and the weight of the larvae were calculated. Hemicellulose had the greatest |*r*| value (0.9569). Further research revealed that larvae raised on hemicellulase-pretreated finishing pig manure frequently weighed 21–30% (days 2–8) more than larvae raised on control manure. In conclusion, hemicellulose was a significant component that might hinder larval growth. The results of this study could be used to improve the system before it is put into use.

## 1. Introduction

The number of enclosed, high-density pig farms has substantially increased in China due to the rapid growth of intensive breeding, which has led to the creation of 1.64 × 10^9^ tons of manure in 2017 [1]. Manure comes from two different types of herds: breeding herds and fattening herds, with fattening herds producing the majority of the pig manure. In China, weaned piglets are raised on the fattening farm in three stages: the nursery period, the growing period, and the finishing period. There is a wide diversity of nutritional requirements throughout the fattening period, resulting in discrepancies in feeds given and manure produced [2,3]. Pig manure contains organic pollutants, heavy metals, and pathogens; is a source of environmental pollution; and poses a public health threat. The control of pig manure pollution focuses on two aspects: the optimization of the rearing process and the technologies used for proper disposal. Multi-objective feed formulation (MOF) has been used to reduce the organic matter in manure in the pig rearing process [4]. Additionally, a variety of manure processing technologies have been developed to alter the material’s physical, chemical, and/or biological characteristics, either minimizing its environmental impact or enhancing its transport. However, despite the wide array of manure processing methods that are available and the promise some of these methods have to lessen the environmental effects of pig manure, these technologies have not been widely implemented in international livestock chains [5].

Insects could make a substantial contribution to manure treatment, in addition to aerobic composting and anaerobic digestion [6]. Insects are gaining attention for their ability to produce protein, fat, and micronutrients while disposing of waste, and in some food impoverished areas, they have been an important source of dietary protein for humans [7]. Although there are many species of edible insects, most of them are difficult to raise and harvest and can only be caught in the wild. The black soldier fly (BSF) has been investigated in recent years because of its wide feeding habits, high conversion rate, environmental friendliness, and low cost [8]. Six larval stages make up a BSF’s life cycle, which ends with the prepupa stage. At this time, the BSF leaves the growth substrate and searches for a dry, secure location where it can pupate (go through the metamorphosis stage) and emerge as an adult, which is regarded as a non-pest insect that can survive without food [9]. Black soldier fly larvae have proven to be useful in processing different livestock manures [5,10,11,12]. It has the potential to provide several advantages simultaneously. In one to two weeks, BSFL can change the physical, chemical, and biological characteristics of manure while also changing the original moisture and nutrient levels [6]. Different studies have shown that the composition of the diet, feed rate, density of larvae, feeding patterns, humidity, and temperature could influence the degree of waste reduction and bioconversion, which are often used to determine conversion effectiveness [13,14,15]. Studies on the midgut, the primary organ involved in digestion, and BSFL feeding studies indicate that protein, non-fiber carbohydrates (NFC), and lipids are highly digestible by BSFL, and as a result, their provision improves performance. In contrast, cellulose and other types of fiber are less easily digested and are likely to slow larval growth [13,15,16]. The MOF of fattening pig feed was modified three times to meet the nutritional requirements of the nursery, growing, and finishing pigs. As a result, it should be investigated whether these different diets cause nutritional changes in the produced manure, as well as the effects of such changes on BSFL growth. So far, the literature on this issue is rare. In addition, corn–soybean-based swine diets are currently popular in China [17]. Therefore, the effective pretreatment of fibers from these crops should also be considered. For instance, *Aspergillus niger*-produced hemicellulase, β-mannanase, and β-1,4 mannanase are effective in the digestion of corn–soybean-based pig diets. [17]. Thus, the hemicellulose in the manure could also be treated with the enzyme from this origin.

This study began by investigating the nutritional composition of fattening pig diets from the Wens foodstuff group, which raises fattening pigs in the majority of the provinces in China. Three manure types were collected from the nursery, growing, and finishing pig groups raised by the Wens foodstuff group with clear background information on the pig ages and feed nutrients. Then, the nutrient compositions of the three manure types were analyzed. Furthermore, a comparative assessment of the growth of *H. illucens* larvae reared on three fattening manures was carried out, and the waste reduction was also calculated. The correlation coefficients (*r*) between larval growth and available nutrients were used to screen for factors that would impede larval growth. The influence factor with the highest |*r*| value was further confirmed with a larval growth experiment. The results based on the representative fattening pig manures can be used to identify the optimal aspects of high-grade insect products used in treating swine manures, and the formulation of waste streams can be further researched to enable better usage of the waste.

## 2. Materials and Methods

### 2.1. Acquisition of Different Fattening Pig Manures

Pig excrement was gathered at a farm in Xinzhou, Shanxi province, People’s Republic of China. Three distinct groups of fattening pigs (nursery pigs, growing pigs, and finishing pigs) of the same breed (Trispecific hybrid) created the manures. The manure was collected during the middle of the three periods, and the pigs were fed with three commercial formulation diets manufactured by the Wens foodstuff group (see Table S1 for the nutrients of the feeds). When the manure was collected, the nursery pigs were 6 weeks old, the growing pigs were 15 weeks old, and the finishing pigs were 22 weeks old. The collected manure samples were freshly excreted; they had made contact with the floor but made less contact with urine. A batch of three sealed 10 L plastic bags containing about 10 kg of each type of manure were collected at each stage and were then brought to Jinzhong University’s facilities. The plastic bags with manure were kept at −21 °C until the start of the experiment.

### 2.2. Composition of the Experimental Biowastes

The following day, using an electric oven set to 65 °C for 24 h, the moisture content of the substrates was determined at the start of the trial using 10 g of each substrate. The pH value was analyzed with a portable pH probe. The gross nutritional composition and moisture content of oven-dried (105 °C) wastes were analyzed. The contents of glucose, starch, cellulose, and hemicellulose were determined in triplicate with commercial kits (Bocui Biotechnical Co., Ltd., Shanxi, China). In brief, glucose oxidase catalyzes glucose and produces hydrogen peroxide; peroxidase catalyzes hydrogen peroxide to oxidize 4-aminoantipyrine coupled with phenols to produce colored compounds; for the starch content, the soluble sugars in the sample can be separated from the starch by using 80% ethanol, and the starch content can be calculated by further decomposing the starch into glucose by acid hydrolysis and determining the glucose content using the anthrone colorimetric method. Cellulose can be decomposed into β-glucose when heated under acidic conditions. β-glucose can be dehydrated under the action of a strong acid to form β-furaldehyde compounds. The β-furaldehydes are dehydrated and condensed with anthrone to produce furfural derivatives. The color of the sample can indirectly quantify the cellulose content. Hemicellulose is converted to reducing sugars by acid treatment, which produces a red-brown substance. The protein was calculated based on nitrogen results determined by a titration assay with hydrochloric acid. Then the protein was calculated by multiplying the nitrogen results by a conversion factor of 4.04 [18]. Finally, the samples were extracted with petroleum ether to completely remove all lipids, and the lipid content was determined by subtracting the weight of the residue from the weight of the specimen. 

### 2.3. Experiment Design 

BSF eggs were obtained from a business (Wuliang Biotechnical Co., Ltd., Guangzhou, China) and incubated with wheat bran in a plastic box before being placed in a climate room to hatch. An environment of 28 °C and 75% humidity was chosen as the hatching conditions. After the eggs hatched, different experiment groups were set up as follows: nursery pig manure (NM), 100 four-day-old larvae cultured in 100 g of nursery pig manure; growing pig manure (GM), 100 four-day-old larvae cultured in 100 g of growing pig manure; and finishing pig manure (FM), 100 four-day-old larvae cultured in 100 g of finishing pig manure. Each group contained three replicates, and a round plastic box (diameter: 9.8 cm; height: 10 cm) was used as a container for each replicate. In addition, the 100 four-day-old larvae for each replicate were weighted to determine the initial average larval weight and to ensure no significant discrepancy within or between groups.

When the larvae were 12 days old, after they had been removed from the medium, they were freeze-dried and weighed. Then, the final average larval weight was determined by dividing the dry weight of all larvae from each replicate by the number of larvae. The mass of total feed and the remaining substrates (after the larvae were removed) were dried at 80 °C in a laboratory oven. Then, the overall dry mass of total feed (feed) and the dry remaining substrates (residue) were weighted in order to assess the larvae’s ability to ingest and metabolize the growing substrates. The waste reduction (WR) in the dry matter (DM) indicates the larvae’s ability to reduce feeding substrates. Higher values indicate a larger ability to reduce the organic matter. The equation was computed as follows [19]:

(1) $$\mathrm{Waste}\;\mathrm{reduction}\;(\%\;\mathrm{DM})=\left(1-\frac{\mathrm{residue}\;(g)}{\mathrm{feed}\;(g)}\right)\times 100\%$$

### 2.4. The Pretreatment of Finishing Pig Manure with Hemicellulase

Hemicellulase (of *Aspergillus niger* origin) was obtained from the commercial company Solarbio Life Sciences (Beijing, China). The FM was pretreated with hemicellulase before further BSFL feeding. The enzyme digestion conditions were similar to the previous study [20]. In brief, the manure was dissolved in a CaCl_2_ solution (8 mmol L^−1^) at a ratio of 1:2, a temperature of 50 °C, enzyme addition of 40 U g^−1^ substrate, and an enzymatic digestion time of 60 h to ensure the hemicellulose was completely decomposed. The control group was treated with the same steps as above, except that no enzyme was added. The 4-day old larvae were added to the experimental group and the control group, and the initial average weight of the larval control group was as previously described. After the trial started, the larval weight was measured by selecting 10 of the largest larvae every two days until the larvae were 12 days old and freeze-drying them.

### 2.5. Statistical Analysis

A one-way ANOVA and a least-significant difference (LSD) multiple comparison test were used to assess the statistical significance of differences among treatments and were analyzed using SPSS version 25.0 software (IBM Corp., Armonk, NY, USA).

The Pearson correlation was used to identify the linear dependencies by comparing the glucose, starch, cellulose, hemicellulose, protein, and lipid content of different fattening pig manures with the final average larval weight. A paired t-test was used to analyze the difference in weight between larvae reared on manures pretreated with hemicellulase and larvae in control manures. Statistics were performed using GraphPad prism software (GraphPad Software Inc., San Diego, CA, USA).

## 3. Results

### 3.1. Different Fattening Manure Compositions

The nutrient compositions of the different fattening pig manures are shown in Table 1. The protein composition of the biowastes varied greatly (F_2,6_ = 4637.881 *p* < 0.0001), with FM having the lowest protein content and NM having the highest. Hemicellulose, another component with significant variations (F_2,6_ = 40.29 *p* = 0.0003), was found to be present in varying amounts in the three manure types, with FM having the highest concentrations and NM having the lowest.

Different manures had low levels of total NFC, glucose, starch, and lipids. The lipids and starch of various manures were nearly the same. Additionally, there was no significant variation in the NFC content between the groups. The protein-to-carbohydrate ratios in the manures in the current study did not reach 1:1 due to poor NFC. In comparison to NM, the cellulose content of the growing and finishing manures was virtually identical and substantially greater.

### 3.2. Larval Performance with the Different Pig Manures

**Figure 1:**
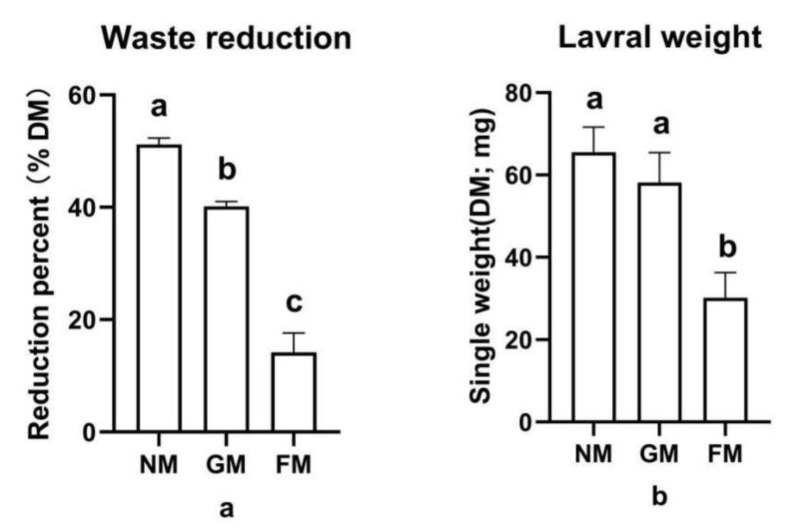
The waste reduction and weight of larvae fed with different fattening pig manure types: (**a**) waste reduction of different groups; (**b**) larval weight of different groups. a, b, c: results with no shared letter are significantly different from each other.

The high waste reduction from using GM and NM was 26% to 37% greater than the use of FM (Figure 1a), and a significant trial effect was found (F_2,6_ = 236.853; *p* < 0.0001). Additionally, a comparison of the larval weights across the three types of manure shows a similar trend to the waste reduction results, and a significant effect was also found in general (F_2,6_ = 4.337; *p* = 0.0013); however, there was no significant difference in the larval weight between NM (65.5 ± 6.2 mg) and GM (58.2 ± 7.3 mg). Larvae given FM had the lowest weight (30.2 ± 6.1 mg) (Figure 1b). With regard to waste treatment and larval biomass production, NM (WR: 51.2%; larval biomass: 65.5 ± 6.2 mg) and GM (WR: 40.2%; larval biomass: 58.2 ± 7.3 mg) outperformed FM (WR: 14.2%; larval biomass: 30.2 ± 6.1 mg), which performed poorly in both areas.

### 3.3. The Correlation between Nutrients and Larval Weight

**Figure 2:**
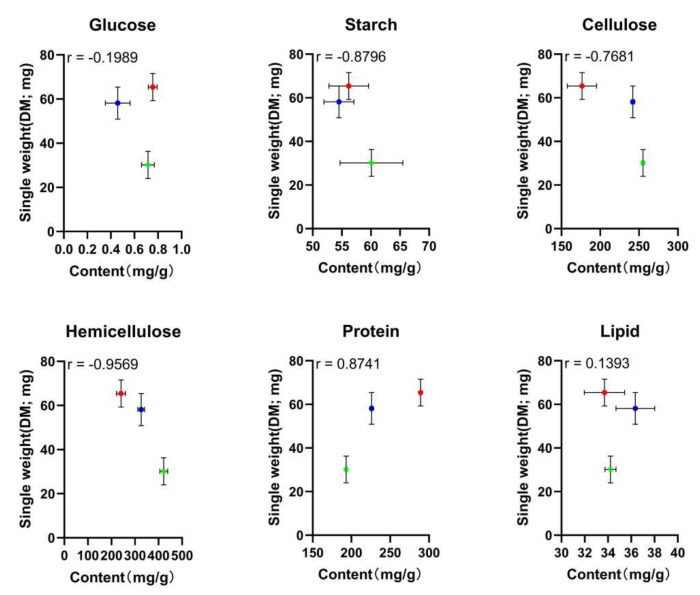
Correlation between the larval single weight and the different nutrient contents. Each point represents the larval single weight of one type of manure plotted against the nutrient content. The *r* value represents the correlation coefficient; a red circle represents the NM; a blue circle represents the GM; a green circle represents the FM.

The correlation coefficients (*r*) between manure nutrients and larval weight were determined, as shown in Figure 2. The |*r*| value for hemicellulose was 0.9569, which is higher than that for glucose, starch, cellulose, protein, and lipid (0.1989, 0.8796, 0.7681, 0.8741, and 0.1393, respectively). In general, when the |*r*| value > 0.4000, the correlation is considered relatively strong. Thus, the correlation was relatively strong between the contents involving starch, cellulose, hemicellulose, protein and the larval weight. The |*r*| value for hemicellulose was greater than those for the other nutrients.

### 3.4. Hemicellulose Influence on Larval Growth

**Figure 3:**
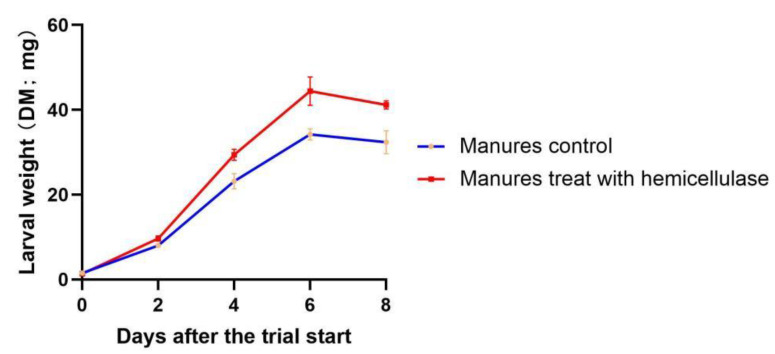
Dry weight of black soldier fly larvae fed hemicellulase-treated manure and control manure.

Because FM had a higher amount of hemicellulose than the other two types, the influence of hemicellulose on larval growth was further studied with FM. The results of days 2–8 revealed that larvae reared on manures pretreated with hemicellulase often weighed 21–30% more than larvae in control manures (Figure 3), and the paired *t*-test revealed significant differences (*p* = 0.0374). Larvae raised on both types of manures attained their maximum weight on day six.

## 4. Discussion

BSFL have been effectively used to treat a variety of biowastes, including animal manure [12], food waste [12], agricultural residues [21], fecal sludge [22], and so on. Scala et al. reared BSFL using apples and bananas as feed, alone or mixed, and found that the waste reduction reached 59–64% and the total weight gain of larvae reached 882–1052 g [23]. Fermented corn stover was used to feed BSFL, and the waste reduction was up to 48.41% [24]. Li et al. fed BSFL with a combination of soybean residue and food waste in different ratios, and the waste reduction for different groups was 32.71–58.36% [25]. Gold et al. evaluated the larval weight and waste reduction after feeding BSFL various biowastes (mill byproducts, canteen waste, human feces, poultry slaughterhouse waste, cow manure, and vegetable canteen waste). The results showed that the range of the larval weight was 14.3–59.1 mg (dry weight), and the range of the waste reduction was 12.7–67.7% [13]. Miranda et al. fed pig manure to the larvae, and the larval weight reached 150 mg (wet weight) [26]. Normally, one piglet consumes about 300 kg of feed in the growth stage of the nursery-finishing process in China. The consumption of fattening pigs as food is generally accepted around the world, which has led to a sizable number of fattening pigs. Thus, fattening pigs are not only the main feed consumers but also the main manure producers. Considering this, a precise and in-depth study of the association between swine manure and larval growth can contribute to effective swine manure treatment with BSFL.

The results from this study demonstrate that different types of manure from fattening pigs can impact the production of BSFL and the larvae’s waste reduction ability. The highest weight and waste reduction were shown in larvae given NM, while the lowest results were seen in larvae fed FM. In terms of differences, variations in nutritional composition, notably protein and hemicellulose concentration, were discovered across the three manure types. A correlation analysis further supported the relationship between larval growth and both hemicellulose and protein content. It is worth noting that the data also showed a significant association between starch and cellulose based on |*r*| values. The cellulose correlation result, however, disagreed with the content data, which showed no appreciable differences between GM and FM. The starch content of the various types of manure did not differ much, and the *r* value was negative, which was inconsistent with previous findings [27]. Manures had low levels of total NFC, glucose, and starch overall. This was expected because the majority of NFC is digested or fermented in both human and animal guts, and animal tissue only stores extremely modest amounts of glycogen [13,28]. Previous research indicated that a more balanced nutrient profile in wastes was useful for larval growth [29]. Thus, the P:NFC ratio of the manures was also calculated. The results revealed that the nutrients in the manures were not balanced. Interestingly, NM had the greatest nutritional imbalance despite having the best performance in larval growth. Thus, this suggests that NFC is less important for larval development than other nutrients in this study.

The influence of protein content on larval growth has been validated in many studies. For instance, Lalander et al. found that protein has the biggest effect on how quickly a BSF larva develops into a prepupa [14]. Additionally, Beniers and Graham found that protein is more crucial to larval weight than NFC [30]. Waste with a higher protein content can also enhance the lipid content of larvae because amino acids enable larvae to advance to the following instar [31]. According to studies on the common fruit fly larvae (*Drosophila melanogaster*), fly larvae can regulate their protein intake but may overfeed on other nutrients, such as NFC [32]. This emphasizes even more how crucial protein is for growth. When BSFL receive low-protein, high-carbohydrate meals, the presence of protein may have an impact on the weight and fat content of the larvae [33]. In our investigation, larval weight and protein content were closely associated. FM had the lowest protein content, which resulted in the lowest larval weight. Based on past research and our study, it is possible to surmise that protein content is a crucial element in the growth of larvae fed fattening pig manures. However, this needs to be confirmed by further research, particularly in the area of waste formulation.

Cellulose, hemicellulose, and pectin are always plentiful in fiber, which is often characterized as a complex and highly changeable component of plant-based feedstuffs. While hemicellulose and pectin include sugar side chains that make them more easily degradable, cellulose is present in plants in tightly bound aggregates. Fiber had a bad correlation with the rate of bioconversion of biowaste formulations and waste reduction [13]. There have been numerous strategies tested to address this. For instance, it was shown that larvae fed a mixture of rice straw (30 weight percent) and kitchen garbage (70 weight percent) for 10 days were able to convert 27.9% of the cellulose and 32.6% of the hemicellulose [34]. Rid-X, a patented combination of enzymes and bacteria, was added to the conversion system at 0.35 weight percent, and this allowed for the conversion of 65.5% of the cellulose and 56.3% of the hemicellulose [35]. The physicochemical structure and characteristics of rice straw were altered as a result of the removal of a significant amount of lignin and a small amount of hemicellulose using an alkaline peroxide pretreatment. This made it easier for larvae to digest rice straw and boosted their effective conversion rate, especially as cellulose was more readily broken down [36]. On the other hand, researchers have found that BSFL can bioconvert wastes and by-products with a high fiber content because they include cellulase enzymes [37]. The gut microbiota of *H. illucens* contained cellulase, according to Lee et al. [38]. This shows that during the BSFL treatment, some fibers, most likely hemicelluloses, were broken down. In BSFL treatment with artificial diets, Gold et al. also noted some hemicellulose decomposition [37], albeit to a considerably lesser extent than that described by Rehman et al. with cow manure [39]. When formulating biowaste based solely on the glucose and starch content of NFC, such variances in digestibility may produce unexpected performance results because they have not yet been taken into account [13]. There is not much research on how the fiber in pig manure affects BSFL growth. The cellulose in the FM used in the correlation studies did not have a critical role in the growth of the larvae. When cellulose content was evaluated between growing and FM (242.02 mg g^−1^ vs. 255.03 mg g^−1^), there was no discernible difference, while the difference in larval weight growth (DM) is nearly twice as great (30.2 ± 6.1 mg/single larva fed FM vs. 58.2 ± 7.2 mg/single larva fed GM). Additionally, the weight of the larvae given GM was comparable to that of the larvae fed NM (65.5 ± 6.2 mg/larva). These findings showed that, at least for fattening pig feces, cellulose is not the main barrier for larval growth. The correlation coefficients (*r*) suggested that hemicellulose might be crucial for larval weight; however, the correlation test’s P value of 0.1877 indicates that the finding is not statistically significant. Thus, a subsequent digestion test using finishing pig manure with added hemicellulase was conducted. All the procedures and materials employed in the control and enzyme-treated groups were the same because manure is a complicated mixture, with the exception of the addition of hemicellulase in the enzyme-treated group. Larval weights did differ between the control and treatment groups, according to the data from the trial (Figure 3; *p* < 0.05 for 2–8 days).

It is noteworthy that, at day eight, the weight increase in the larvae fed FM with enzyme pretreatment (41.2 ± 1.9 mg/larva) was lower than the weight increase in the larvae fed NM and GM (65.5 ± 6.2 mg/larva and 58.2 ± 7.3 mg/larva, respectively). It might be inferred that influencing variables other than hemicellulose existed in the FM. The protein composition of our experiment could also have a significant impact. The times for manure collection were also carefully selected because the fattening pigs were fed three different types of swine formulation feed (Table S1) during the nursery, growing, and finishing periods, respectively. Thus, the manure was collected in the midst of each of these periods, which could ensure that the manure is representative of each stage. The corn–soybean-meal-based diets are widely used for livestock in China, especially in fattening pig farms. For these reasons, future studies should investigate the effect of the formulation of feed diets on fattening pig manure to promote the growth of BSFL.

## 5. Conclusions

The current study highlights the differences in larval weight and waste reduction for larvae fed different types of fattening pig manure. According to the manure’s nutrient composition and the correlation analysis, the hemicellulose content might be a crucial influencing factor. Further experiments confirmed the influence of hemicellulose on larval weight. Additionally, this study also shows the protein content differences that are likely to exist across different manure types because the pigs in different growth stages were fed various feeds. These findings suggest that future research on using pig manure as a BSFL substrate should carefully take the composition of the nutrients into account. The relation between the substrate’s formulation and larval development performance should be further studied at a larger scale to advance the industrialization of BSF, which will contribute to a more circular and climate-neutral society.

## Figures and Tables

**Table 1:** Nutrient composition of different fattening pig manure types.

Manure Types	pH	Moisture Content (%)	Proteins (mg g^−1^)	Lipids (mg g^−1^)	Non-Fiber Carbohydrates (mg g^−1^)		Fiber (mg g^−1^)		P:NFC *
					Starch	Glucose	Cellulose	Hemicellulose	Ratio
NM	5.7 (0.0)	68.4 (0.3)	289.21 (0.85) ^a^	33.70 (1.73) ^a^	56.19 (3.39) ^a^	0.76 (0.04) ^a^	176.44 (18.82) ^b^	240.97 (19.34) ^c^	0.2090
GM	5.6 (0.0)	67.7 (0.6)	225.97 (1.76) ^b^	36.36 (1.66) ^a^	54.50 (2.59) ^a^	0.46 (0.10) ^b^	242.02 (1.99) ^a^	326.73 (14.36) ^b^	0.1674
FM	6.2 (0.1)	64.5 (0.0)	193.13 (0.90) ^c^	34.22 (0.48) ^a^	60.08 (5.41) ^a^	0.72 (0.05) ^a^	255.03 (1.01) ^a^	422.44 (16.42) ^a^	0.1257

Note: In parenthesis is standard deviation for samples; The superscript letters (a, b, c): results with no shared letter are significantly different from each other; * P:NFC = ratio of protein to non-fibre carbohydrates (NFC).

## Data Availability

Summarized data are presented and available in this manuscript and the rest of the data used and/or analyzed are available from the corresponding author on reasonable request and pending agreement by co-authors.

## Supplementary Materials

The following supporting information can be downloaded at: https://www.mdpi.com/article/10.3390/ani13030452/s1, Table S1. The nutrients of nursery-finishing pig formulation diets.
