# Supplementary material for: Assessing Nursery-Finishing Pig Manures on Growth of Black Soldier Fly Larvae
Source: Animals (Basel). 2023 Jan 28;13(3):452. doi: 10.3390/ani13030452 (PMC9913757; doi:10.3390/ani13030452)
Supplement: Supplementary file 1 [file animals-13-00452-s001.zip › animals-2124366-supplementary.pdf]

**Supplementary materials**

**Table S1** The nutrients of nursery-finishing pig formulation feeds.

| | Dry matters (%) | Water (%) | Proteins (%) | Lipids (%) | Fiber (%) | Energy (kcal $kg^{-1}$) |
|---|---|---|---|---|---|---|
| NPFF | 87.51 | 12.49 | 16.98 | 4.0 | 12.32 | 2479 |
| GPFF | 87.30 | 12.70 | 15.02 | 2.6 | 12.68 | 2499 |
| FPFF | 87.22 | 12.78 | 12.50 | 2.9 | 13.45 | 2530 |

Note: NPFF: nursery pig formulation feed; GPFF: growing pig formulation feed; FPFF: finishing pig formulation feed.
